# Antibiotic Exposure Leads to Reduced Phage Susceptibility in Vancomycin Intermediate Staphylococcus aureus (VISA)

**DOI:** 10.1128/aac.02247-21

**Published:** 2022-06-16

**Authors:** Shawna McCallin, Carmen Menzi, Swenja Lassen, Jean Daraspe, Frank Oechslin, Philippe Moreillon

**Affiliations:** a Department of Fundamental Microbiology, University of Lausanne, Lausanne, Switzerland; b Department of Neuro-Urology, Balgrist University Hospital, University of Zürich, Zürich, Switzerland; c Electron Microscopy Facility, University of Lausanne, Lausanne, Switzerland

**Keywords:** bacteriophage, phage therapy, antibiotic resistance, *Staphylococcus aureus*, vancomycin, VISA, antimicrobial resistance, staphylococcus

## Abstract

In the time of antimicrobial resistance, phage therapy is frequently suggested as a possible solution for such difficult-to-treat infections. Vancomycin-intermediate Staphylococcus aureus (VISA) remains a relatively rare yet increasing occurrence in the clinic for which phage therapy may be an option. However, the data presented herein suggest a potential cross-resistance mechanism to phage following vancomycin exposure in VISA strains. When comparing genetically similar strains differing in their susceptibility to vancomycin, those with intermediate levels of vancomycin resistance displayed decreased sensitivity to phage in solid and liquid assays. Serial passaging with vancomycin induced both reduced vancomycin susceptibility and phage sensitivity. As a consequence, the process of phage infection was shown to be interrupted after DNA ejection from adsorbed phage but prior to phage DNA replication, as demonstrated through adsorption assays, lysostaphin sensitivity assays, electron microscopy, and quantitative PCR (qPCR). At a time when phage products are being used for experimental treatments and tested in clinical trials, it is important to understand possible interference between mechanisms underlying antibiotic and phage resistance in order to design effective therapeutic regimens.

## INTRODUCTION

Staphylococcus aureus causes a multitude of infections in both humans and animals, ranging from superficial skin and wound infections to severe deep-seated abscesses and sepsis, including life-threatening endocarditis (1, 2). This facultative pathogen has developed resistance to multiple antibiotics, thereby making infections difficult to treat and leading to increasing hospital costs and mortality rates (3, 4). The prototype is methicillin-resistant S. aureus (MRSA), where an alternative penicillin-binding protein (PBP), PBP2A, which has a greatly reduced binding affinity for β-lactams, mediates resistance to this entire class of antibiotics (5, 6). While MRSA infections are often treated with the glycopeptide antibiotic vancomycin, resistance to vancomycin has been detected since the late 1990s (7–9).

Several categories and mechanisms of glycopeptide resistance exist (10, 11). Vancomycin-resistant S. aureus (VRSA) strains have acquired the *vanA* gene cluster through horizontal gene transfer and display low sensitivity (i.e., high minimum inhibitory concentrations (MICs) of >32 μg/mL) of vancomycin due to resulting reduced affinity (12). Other strains, referred to as vancomycin-intermediate S. aureus (VISA), characteristically display thickened cell walls, decreased growth rates, and elevated MICs of vancomycin ranging from ~2 to 16 μg/mL (13) resulting from impaired diffusion of the antibiotic to its site of action at the cell membrane (10, 14). Stepwise accumulation of mutations in several genes result in a progressively more pronounced VISA phenotype, but the number of mutations, implicated genes, and sequential order of mutation acquisition remain undeciphered, and it is likely that there are numerous combinations that progressively give rise to VISA. Preceding the establishment of VISA is a state in which cell subpopulations of the strain possess various degrees of the implicated mutations, which is referred to as heterogenous VISA (hVISA). VISA and hVISA traits facilitate the establishment of persistent infections and have been associated with an increased risk for treatment failure, especially in biofilm-mediated bone and joint infections (BJIs) (15, 16).

New treatment options are needed to combat antibiotic resistance and infections such as those caused by VISA or VRSA, one of which is bacteriophage (phage) therapy (17). Phages are viruses of bacteria and their lytic activity against bacterial hosts can be exploited therapeutically to treat infections that cannot be controlled by antibiotics or that are biofilm-related. Lytic phages first adsorb to host bacteria through specific surface receptors, eject their DNA into the bacterial cytosol, then reprogram the host cell for phage production, and eventually lyse their host bacterium to release their progeny. Phage therapy has been used in several countries, such as Russia, Georgia, and Poland, for many years, and numerous commercially available phage products contain phages against S. aureus (18–21). While investigating these products in the context of a phase l clinical trial, we previously observed that staphylococcal phages from commercial products had reduced activity against VISA strains (22). Other researchers have observed direct (23) or indirect (24) associations between the VISA phenotype and reduced phage sensitivity. To explore cross-resistance against vancomycin and phages as a proof of concept, we tested several vancomycin-susceptible S. aureus (VSSA) and VISA strains against phage K and tested the effect of vancomycin exposure on different steps of phage infection in the background of clinical S. aureus isolate PC3 (25, 26).

## RESULTS

### Phage activity is reduced in VISA strains.

**FIG 1:**
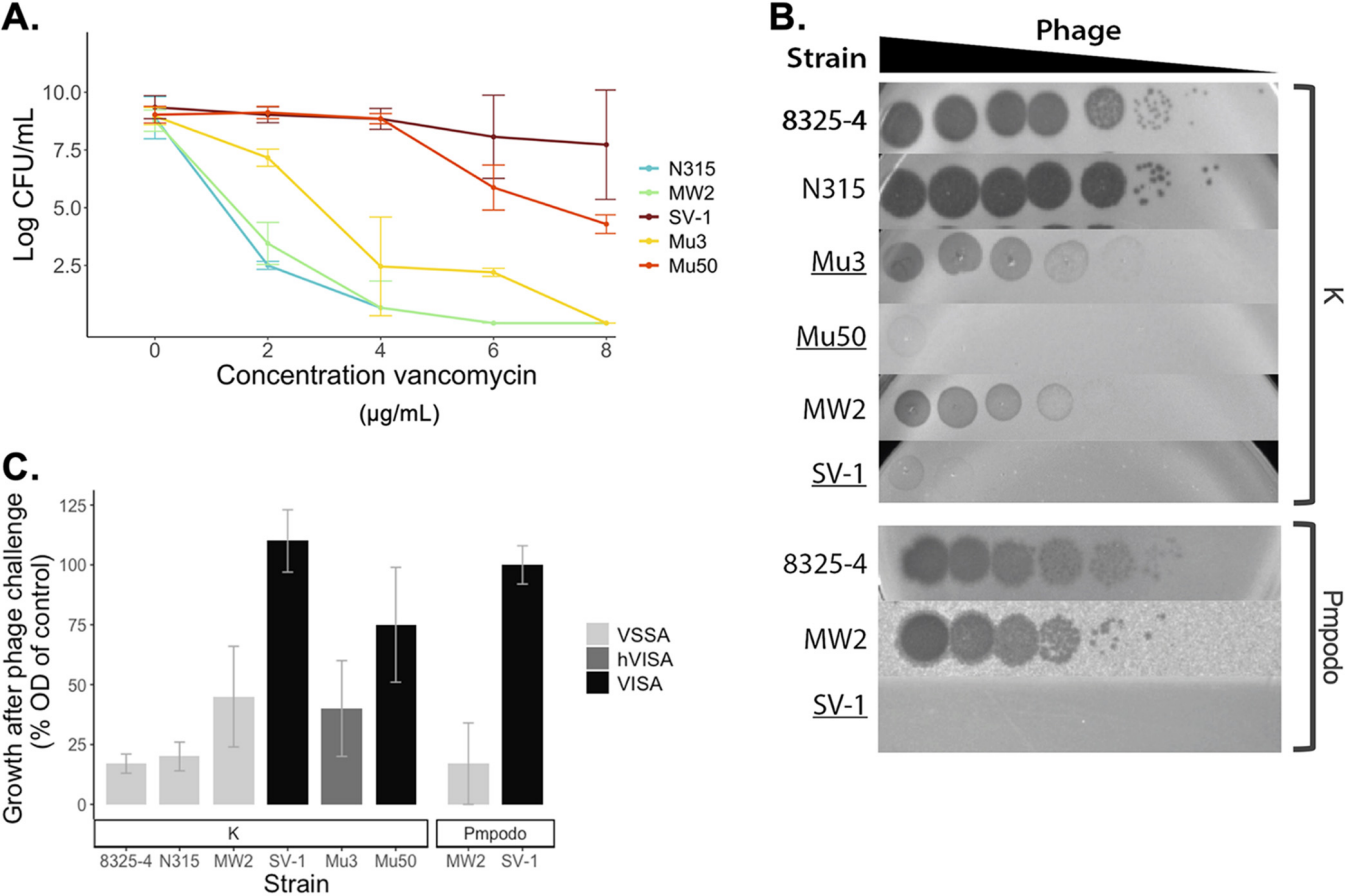
Phage sensitivity of strains displaying different levels of resistance to vancomycin. (A) Vancomycin population analysis profiles of selected strains. (B, C) Activity of phages K or Pmpodo on indicated strains as shown through dilution spot assays (B), in which the hVISA and VISA strains are underlined, and turbidity reduction assays at 4 h postinfection (C). The experiments were performed in triplicate. OD, optical density; VISA, vancomycin-intermediate S. aureus; hVISA, heterogenous VISA; VSSA, vancomycin-susceptible S. aureus.

To determine whether phage sensitivity differed between VSSA and VISA isolates, bacteria were challenged with the broad-host-range myovirus phage K (Fig. 1). Broth microdilution according to EUCAST guidelines was used to confirm vancomycin susceptibilitiy: VSSA strains 8325-4, MW2, and N315 were sensitive to concentrations of vancomycin of <2 μg/mL, while the MIC for hVISA strain Mu3 was 2 μg/mL, and VISA strains Mu50 and SV-1 persisted at >2 μg/mL (Table 1). Population analysis profiles were performed to identify subpopulations with increased resistance to vancomycin (Fig. 1A). SV-1 is a laboratory-derived VISA mutant of the VSSA strain MW2 (27), making these two strains a useful isogenic strain pair to study VISA-associated phenotype changes in relation to phage infection. The strains hVISA Mu3 and VISA Mu50 were both isolated in Japan (9, 28), share >99% sequence identity, and have nearly identical phenotypes except for their degree of vancomycin susceptibility. Using such strain pairs, it was possible to compare phage activity in strains with shared genetic backgrounds.

**TABLE 1:** Bacterial strains^*a*^

Strain	Parent	Accession no.	Antibiotic profile	MIC (μg/mL)	Reference
8325-4	8325	CP000253 ^*b*^	MSSA	1	64, 65
Mu3		NC_009782	hVISA	2	28
Mu50		NC_002758	VISA	4	9
PC3		NA	VISA	4	25
PC3*	PC3	NA	VISA	8	25
PC3*van−	PC3*	NA	VSSA	1	This study
PC3*van+	PC3*	NA	VISA	8	This study
MW2		NC_003923	MRSA	1	27
SV-1	MW2	NA	VISA	16	27
N315		NC_002745	MRSA	0.5	53
VRSA collection			VRSA	>16^*c*^	BEI Resources

a The table shows the bacterial strains and parental relationships used in this study. The MICs to vancomycin were determined by broth microdilution according to EUCAST guidelines. MSSA, methicillin-sensitive S. aureus; MRSA, methicillin-resistant S. aureus; hVISA, heterogenous vancomycin-intermediate S. aureus; VISA, vancomycin-intermediate S. aureus; VRSA, vancomycin-resistant S. aureus; NA, not available.

b Parent strain sequence.

c Not tested experimentally within this study.

Bacteria were challenged with phage and observed for evidence of phage infection (i.e., number, size, and clarity of plaques in plaque assays or turbidity reduction of liquid bacterial cultures). In dilution spot assays (Fig. 1B), phage K produced similarly high titers (dilution, ≥10^−6^) on VSSA strains 8325-4 and N315 and, to a lesser degree, on VSSA strain MW2 (dilution, 10^−4^). In contrast, VISA strains (Mu3, Mu50, and SV-1) showed decreased or no susceptibility to phage K. Interestingly, while phage K was able to produce plaques on hVISA strain Mu3, the observed plaques were visibly smaller than VSSA strains (Fig. S1). Due to the lower sensitivity of VSSA strain MW2, phage infectivity with the genetically unrelated podovirus, Pmpodo, was performed, and a complete loss of phage sensitivity was also observed between the MW2 and SV-1 strain pair, thus suggesting that this phenomenon may not be specific to phage K (Fig. 1B and C). These findings are further supported by efficiency-of-plating (EOP) (Table 2). Since the dynamics of phage-bacteria interactions can be different in solid versus liquid media, sensitivity was also tested in turbidity reduction assays (Fig. 1C). In the presence of phage at 4 h postinfection, the turbidity of VSSA cultures was reduced to between 8 and 45% of noninfected cultures, whereas the turbidity of VISA cultures ranged from 41 to >100% of control growth. The difference was clearer when specifically comparing growth within the strain pairs having highly similar genomes except for mutations related to vancomycin resistance (i.e., Mu3 versus Mu50 or MW2 versus SV-1).

**TABLE 2:** Efficiency-of-plating (EOP) values for indicated strains and phages^*a*^

	EOP	
Strain	K	Pmpodo
8325-4	1.00	1.00
N315	1.04	–
Mu3	0.58	–
Mu50	0.00	–
MW2	0.71	0.10
SV-1	0.00	0.00

a –, not evaluated.

### Vancomycin exposure results in decreased phage sensitivity.

**FIG 2:**
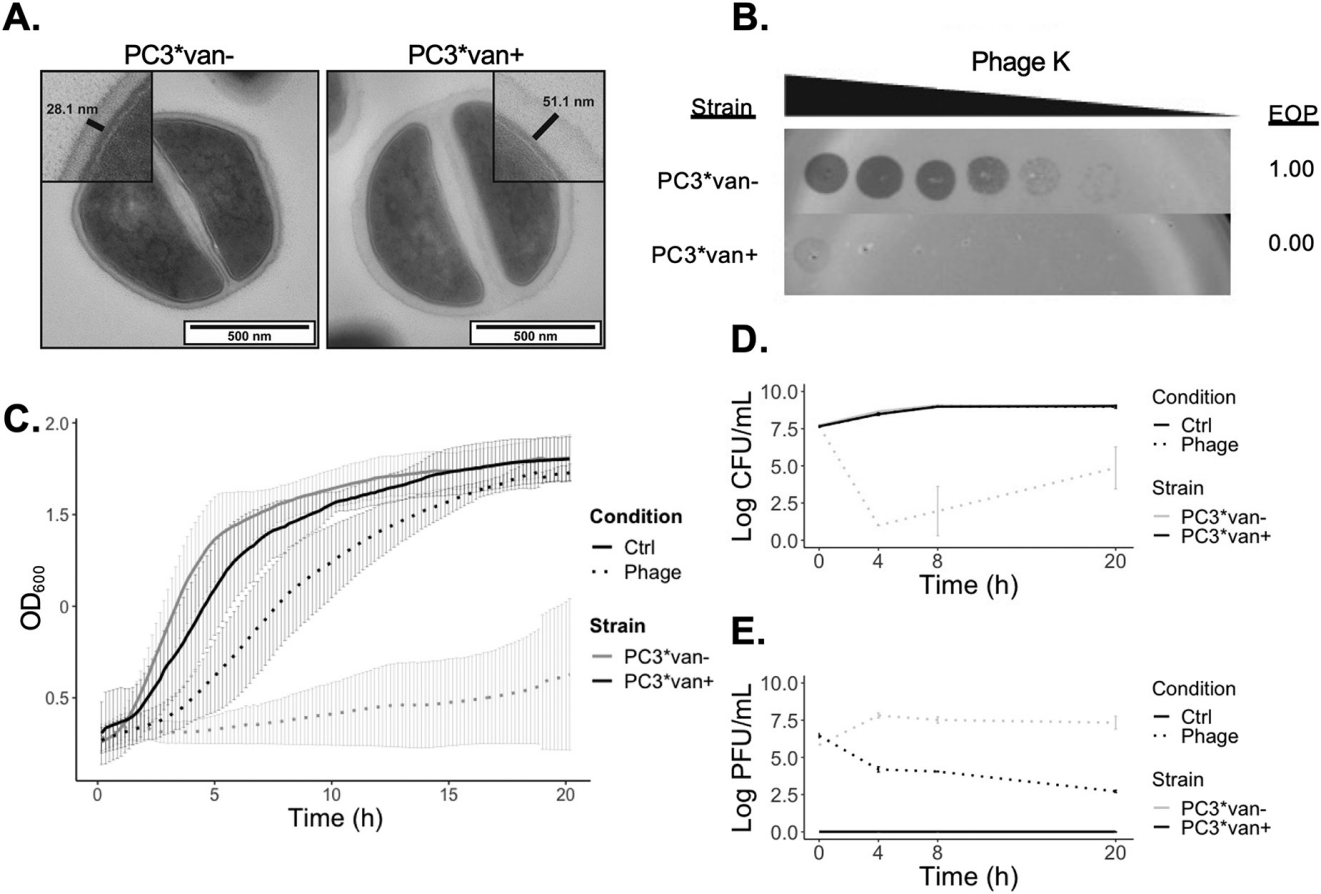
Effect of vancomycin exposure on cell morphology and phage infectivity. (A) Transmission electron microscopy (TEM) of PC3* after growth in antibiotic-free media (PC3*van−) or media containing vancomycin (PC3*van+). The insets show cell wall measurements (not drawn to scale). (B) Dilution spot assays of phage K and efficiency of plating (EOP) values. (C) Growth curves of PC3*van− (gray) and PC3*van+ (black) in the presence (dotted line) or absence (straight line) of phage K over time as measured by optical density at 600 nm (OD_600_). The bars correspond to standard deviation of five replicates. (D) Viable bacterial counts at the indicated time points. (E) Phage counts at the indicated time points.

In order to further determine whether phage susceptibility differs or can be exacerbated in relation to vancomycin exposure, the clinical background of PC3, a patient isolate whose treatment failure was suspected due to increased vancomycin resistance, was used (Table 1) (29). Under laboratory conditions, PC3*, a derivative of PC3 in the original study, was exposed to further vancomycin pressure here and was serial passaged in vancomycin-free (van−) or vancomycin-supplemented media (van+) in order to revert or induce the VISA phenotype (29). For the strain passaged with vancomycin (PC3*van+), a physical thickening of the cell wall following vancomycin exposure was observed by transmission electron microscopy (TEM) compared to the strain resulting from passaging without vancomycin (PC3*van−; Fig. 2A). Cell wall thickness is a characteristic feature of VISA strains (25, 27, 29). Cell wall thickness measured 50.3 ± 7.8 nm for van+ cells compared to 29.0 ± 4.8 nm for van− cells. This difference in cell wall thickness reflected a difference in MIC to vancomycin of 8 versus 1 μg/mL for PC3*van+ and PC3*van−, respectively. Exacerbation of the VISA phenotype in PC3*van+ compared to PC3*van− enabled a comparison of phage activity in the same genetic background.

Phage sensitivity was markedly decreased for PC3*van+ compared to PC3*van−, thus showing that phage resistance can be inducible by vancomycin exposure (Fig. 2B and C). Dilution spot assays also showed that phage sensitivity differed from the parent strains PC3 and PC3* (Fig. S2) (29). In turbidity reduction assays, the difference in phage sensitivity was maintained over 20 h and evidenced through optical density and viable counts of phage and bacteria (Fig. 2C to E). Phages were also tested against a panel of eleven VRSA strains, four of which displayed decreased phage sensitivity. However, after strains were serially passaged in the presence or absence of vancomycin, no change in phage sensitivity was observed, therefore indicating that this altered phage sensitivity is specific for VISA-related changes rather than to vancomycin itself (Table S1).

### Vancomycin exposure interferes with phage DNA replication.

**FIG 3:**
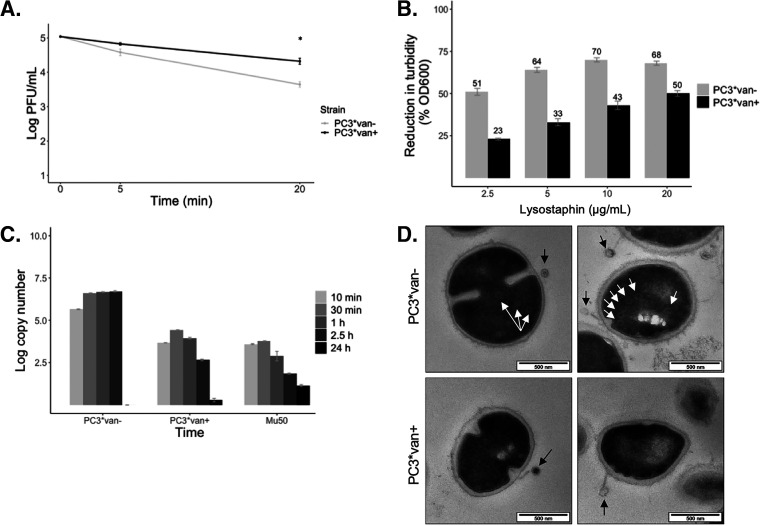
Measurement of the phage infection processes in strains with and without vancomycin exposure. (A) Adsorption assay of phage K at 5 and 20 min postinfection as measured by viable titer (log_10_ PFU/mL). *, *P* < 0.01. (B) Turbidity reduction after 10 min at different concentrations of lysostaphin (% loss initial OD_600_). (C) Quantitative PCR (qPCR) for phage K of indicated strains over time (log_10_ copy number). (D) TEM of phage-infected cultures at 20 min postinfection. White arrows point to intracellular capsids (progeny virions). Black arrows indicate extracellularly absorbed phage.

In order to identify at what stage phage infection may be interrupted in VISA strains, a series of experiments were performed with the vancomycin-exposed or -unexposed strains, PC3*van+ and PC3*van−. The ability of phage K to adsorb to the host surface was confirmed by adsorption assays (Fig. 3A). No significant difference was observed for phage adsorption at 5 min postinfection (*P* > 0.01), when 90% of phage was already adsorbed and was similar to what has been determined for phage K in a previous study (30). The difference in free phage at 20 min was significant (*P* < 0.01); however, phage concentrations continued to further decrease from the media in both cultures after phage addition until ~30 min (Fig. S3). An increase in the concentration of free phage was detected for the PC3*van− strain after 30 min, which indicated the release of newly synthesized progeny phage, and the final phage titer was 100× higher than the initial phage inoculum after 24 h. The titer of phage in culture with the PC3*van+ strain steadily decreased over time and was 3 log_10_ lower than the initial titer after 24 h. Phage receptors therefore remained functional after vancomycin exposure and were not affected by the cell wall modification induced with the VISA phenotype, although adsorption occurred slower to van+ than van− cells (Fig. 3A).

Considering the thickened cell wall of PC3*van+, it was possible that the tail-associated muramidase enzyme (TAME) of phage K was insufficient to degrade the peptidoglycan in order to reach the cell membrane. Lysostaphin is an antibacterial enzyme that, like TAME, targets the pentaglycine interpeptide bridges in the peptidoglycan structure of the S. aureus cell wall (31, 32). Lysostaphin assays showed a concentration and time dependency for enzymatic bacterial lysis (Fig. 3B), where PC3*van+ required higher concentrations or additional time to be lysed compared to PC3*van−. While lysostaphin activity is not a direct analog of phage K TAME, increased cell wall thickness was shown not to be a definitive impediment to peptidoglycan degradation and therefore would not be the causative block to phage infection.

In order to detect whether phage DNA reached the intracellular compartment of their host bacteria, the intracellular concentration of phage DNA over time was measured by qPCR (Fig. 3C). Extracellular phage was detached/lysed and DNA degraded following a previously established protocol (33). For phage-infected PC3*van− cultures, intracellular phage DNA was detected as early as 10 min postinfection and increased to a maximum of 10^8^ copies after 2.5 h (no bacteria were left to sample at the 24 h time point). Phage DNA was also detected intracellularly in phage-infected strain PC3*van+ cultures, as well as reference VISA strain Mu50, at 10 min postinfection, but the copy number never exceeded the phage inoculum and gradually decreased over time. Assuming a ribosomal copy number of 5 for both strains (34), the ratio of copy number of phage-to-bacterial DNA was 17-fold higher for in PC3*van− cultures than PC3*van+ at 30 min postinfection, therefore supporting phage replication in the PC3*van− host (data not shown).

**FIG 4:**
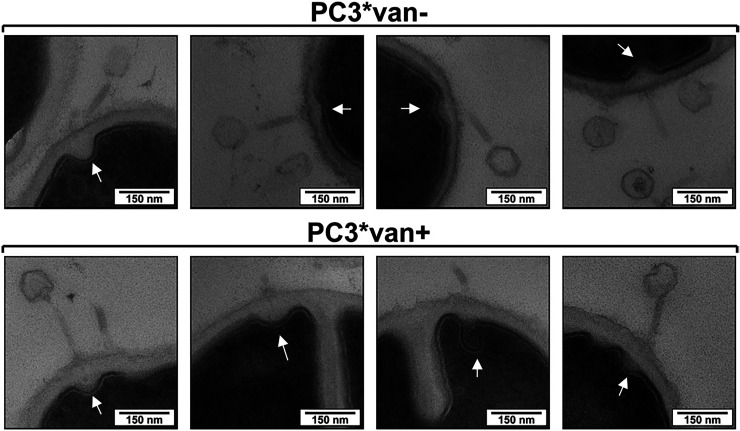
TEM of phage interaction at the cell membrane in strains with (PC3*van+) and without (PC3*van−) vancomycin exposure. Arrows point to cell membrane at the site of phage adsorption and not to the cell septum.

Transmission electron microscopy (TEM) of the phage infection process was done in order to visualize different steps in phage infection (Fig. 3D). In PC3*van−, adsorbed phages mostly with empty capsids (denoted by the light color) were observed, and progeny virions were visible intracellularly 20 min after infection (Fig. 3D, white arrows). For PC3*van+, phages were also observed absorbed to the surface of bacteria, but no intracellular phage particles were detected. Interestingly, phage capsids were also frequently devoid of DNA in PC3*van+, and a systematic uniform random sampling (SURS) on large TEM montages showed that there were no significant differences between the capsid state (empty or full) of phages adsorbed to either strain (*P* > 0.1; Fig. S4). Invagination of the cell membrane at the site of phage adsorption was observed for both strains, indicating that phage DNA was ejected and an influence was exerted on the cell membrane (Fig. 4, arrows). In combination with the previous qPCR data, it appeared that phage DNA was ejected and isolated with the cell fraction, where it was protected from enzymatic degradation prior to DNA extraction.

## DISCUSSION

The first identification of a clinical VISA isolate occurred just over 20 years ago (10, 14), and VISA strains have been associated with an increase in clinical failure, likely due to their ability to create persistent infections through biofilms and/or intracellular residence (35, 36). As alternative treatment strategies are sought to address the shortcomings of such last-line treatments, phage therapy would be a possible candidate. Indeed, phage therapy has been used with apparent success in several case reports for the treatment of recalcitrant infections, including prosthetic joint infection, osteomyelitis, and sepsis (21, 37–39), and shown to be safe in phase l clinical trials (22, 40).

The current study, however, indicates that the lytic life cycle of phage K was impaired in VISA strains, which was induced by prolonged exposure to vancomycin *in vitro*. This proposed vancomycin-phage cross-resistance was first noted as early as 2003 (23), when researchers remarked that VISA strains changed their phage-typing profile but did not investigate the role of vancomycin in phage sensitivity. A more recent publication on the design of an S. aureus phage cocktail for the treatment of antibiotic-resistant infections also showed decreased activity of their phage against a VISA strain panel, infecting only ~64% of strains compared to 94 to 100% of other non-VISA strain panels from different years and geographical locations, including MRSA strains (24). Contrarily, a genome association study did not observe any decreased sensitivity of VISA strains (*n* = 26) to a phage similar to phage K; study differences in terms of growth conditions, the absence of vancomycin pressure, and a high multiplicity of infection (MOI) could account for these findings (41). We previously noted that VISA strains were less sensitive to two commercial phage cocktails from the Eliava BioPreparations (Tbilisi, Georgia), which prompted this current investigation (22).

While previous publications reported the gross observation of phage lysis/no lysis, this investigation documents phage resistance in VISA and dissects out the sequential steps at which phage infection might be blocked. Phage K was primarily used for these studies due to its broad host range and it being closely related to a number of therapeutic staphylococcal phages of Herelleviridae, as well as the well-studied Gram-positive Bacillus phage SPO1 (42, 43). Surface receptors for phage K are located in the backbone of the wall teichoic acid (WTA), and phage adsorption is the first step in the infection process (44). Despite the characteristic thickened cell wall of VISA strains, which we initially hypothesized to be the culprit of decreased phage sensitivity, this did not stop phage adsorption in adsorption assays and was visualized by TEM, indicating that phage receptors retained their functionality. Considering that biosynthetic pathways for WTA are not directly targeted by VISA-related mutations, this may explain why adsorption was still possible. Phage resistance to the closely related Sb-1 phage has been shown to be mediated through poor adsorption for some strains (45), but another study demonstrated that adsorption is still functional in other Sb-1-resistant strains that were not VISA (46).

Another consequence of the thickened cell wall in VISA is a greater amount of peptidoglycan. Compared to VSSA, VISA strains have exhibited altered sensitivity to cell wall-digesting enzymes, such as lysostaphin, or detergents like Triton X (47). In this study, lysostaphin was able to digest thickened cell walls of the VISA strain PC3*van+, thus mimicking the action of phage TAME proteins to tunnel through to the cell membrane. The fact that cell wall thickness was not the major limiting step in phage infection was further supported by TEM, as the physical effects of adsorbed phage on the cell membrane were clearly visible. While the specific mechanism of phage DNA ejection is unknown, membrane convolution at the site of phage adsorption has also been observed for the related Lactobacillus phage LP65 (48).

Intracellular infection steps involve redirection of host metabolism to the purpose of phage DNA replication and protein synthesis. Here, we were able to find evidence of phage DNA entry through qPCR of an arbitrary segment of phage DNA but not of genome replication in VISA strains. Early stages of intracellular infection processes involve shutting down host protein synthesis in order to redirect metabolism to phage replication, and the promoters of early phage genes are recognized by host sigma factors to redirect host RNA-polymerase (RNAP) (49, 50). Further investigating this interaction between early phage genes with the host sigma factors and/or RNAP by RNA sequencing for early phage transcription, Southern blot for phage DNA, or eventually time-resolved fluorescence resonance energy transfer (TR-FRET) for phage-bacteria protein complexes would identify more precisely the infection process that is interrupted in phage infection in VISA.

At this level of granularity, it appears that the infection process is perturbed after DNA ejection and prior to genome replication, which still leaves much to be desired in order to fully understand what derails phage infection in VISA. Part of the difficulty in elucidating this cause is the heterogeneity of genetic mutations and their effector functions, which give rise to VISA. A major study limitation here is the lack of sequencing of strains to determine potential causative mutations, as well as the number of bacterial strains and phages tested. The genetic differences between strain pairs Mu3/Mu50 and MW2/SV-1 shed some light on what mutations may be implicated in phage resistance, indicating that mutations in *vraS* (a two-component regulator), *msrR* (involved with WTA attachment to peptidoglycan), response regulators (*graR* or *walK*), or genes in cellular division and lysis (*ftsZ*, *llM*) or protein synthesis (*rpoC*, *rpoB*, *rpsO*) may play a role, alone or in combination(s) (27, 51–54). Genetic correlation studies with clinical isolates are required and are underway in order to identify certain host mutations that may be involved with decreased phage sensitivity. With this information, it will hopefully be possible to provide insights into the specific mechanisms that are responsible for vancomycin induction of phage resistance.

This study investigated the effect of vancomycin exposure on phage sensitivity and identified a plausible stage during which the phage infection process is interrupted. Furthermore, much like the VISA phenotype, we showed that phage resistance is potentially reversible in VISA strains once vancomycin pressure is removed (29, 55, 56). At a time when phage therapy is heralded as a solution for antibiotic-resistant infections and clinical trials are being planned, it is highly important to anticipate clinical scenarios in which this may prove false. Vancomycin exposure may also compromise clinical outcomes when switching to daptomycin or rifampicin, due to cross-resistance mechanisms between these classes of antibiotics (13, 57). Phage therapy is largely used now only in the case of treatment failure with conventional antibiotics; however, current case reports have not specified the use of vancomycin prior to phage application to know whether therapeutic outcomes could be compromised for VISA infections. The translation of our findings *in vivo* would help to determine the relevance of this study for clinical applications and help to establish effective treatment regimens, such effective combination or administration order of phage and antibiotics.

## MATERIALS AND METHODS

### Bacteria.

The strains used in this study are described in Table 1. S. aureus strains were kept at −80°C in 20% glycerol and cultured at 37°C on brain-heart infusion (BHI) agar plates or in BHI broth with agitation (120 rpm). The media were supplemented with vancomycin as indicated. The strains were kindly provided by Alexander Tomasz (Rockefeller University, New York, NY, USA) and by Baolin Sun (University of Science & Technology of China, Anhui, China). VRSA strains were obtained from BEI Resources (Manassas, VA, USA).

To study the effects of vancomycin exposure on PC3*, 100 μL of the same overnight (ON) broth culture of PC3* was added to 10 mL of BHI with (van+) or without (van−) vancomycin at a final concentration of 2 μg/mL. PC3*van− cultures were serially passaged for 20 days (100 μL into 10 mL fresh BHI without vancomycin). For serially passaged PC3*van+ cultures, vancomycin was increased by 2 μg/mL per day until growth was stable at 8 μg/mL of vancomycin within 24 h; serial passaging at 8 μg/mL vancomycin was then continued for 10 days.

### Antibiotic sensitivity tests.

MICs were determined using the broth microdilution method according to EUCAST guidelines (version 11.0, 2021, www.eucast.org). Briefly, ON cultures were diluted to approximately 1 × 10^6^ CFU/mL and added to an equal volume of antibiotic in Muller-Hinton broth (MHB) supplemented with 0.002% polysorbate 80. Cultures were incubated 37°C and inspected for growth at 24 h.

Population analysis profiles (PAPs) were performed by serially diluting ON cultures in 0.9% NaCl and plating various dilutions on solid agar plates containing increasing concentrations of vancomycin (0, 2, 4, 6, and 8 μg/mL). The results were calculated as the mean viable counts of three independent ON cultures.

### Phage.

The well-characterized and broad host-range myovirus, phage K (kindly provided by Aidan Coffey Munster Technological University, Cork, Ireland), was used for most phage experiments, unless indicated. PMpodo is a phage isolated from the pyophage cocktail (Microgen, Russia), shown to be a podovirus by TEM and previously sequenced, showing 99% identity to phage SCH1 (accession number KY000084), a P68-like virus (22). Phage stocks were prepared by propagating phage on S. aureus strain 8325-4 by inoculating a bacterial culture with an optical density at 600 nm (OD_600_) of 0.2 with a 0.01 to 0.1 multiplicity of infection (MOI) of phage and allowing the culture to incubate for 6 h at 37°C with agitation at which point, the cultures were centrifuged and filtered (0.44 μm). If further concentration was desired, filtered lysates were passed through Amicon ultra centrifugal filters (10-kDa cutoff; Sigma-Aldrich) or precipitated with polyethylene glycol 8000 (PEG; Sigma-Aldrich) at a final concentration of 10% (wt/vol) in 1 M NaCl. Phage pellets were suspended in salt magnesium (SM) buffer (100 mM NaCl, 8 mM MgSO_4_·7H_2_O, 50 mM Tris-Cl at 1 M, pH 7.5) by gentle turning, and PEG was removed with chloroform the next day by mixing 1:1 phage and chloroform, vortexing vigorously, centrifuging for 10 min at top speed, and removing the supernatant. This was repeated until the supernatant was clear. All phages were stored at 4°C, and activity was confirmed on the propagation strain for all experiments.

### Phage activity assays.

Sensitivity to phage was determined using spot tests and double-layer titration, as well as turbidity reduction assays (58). For spot tests, 200 or 500 μL of ON S. aureus culture was mixed with 4 or 10 mL BHI soft agar (BHI supplemented with 7 g/liter of granulated agar; BD Difco) and poured onto standard or square BHI agar plates, respectively. Drops of 3.5 μL of the desired phage dilution were placed on the surface and allowed to dry before incubating ON at 37°C. The plates were observed the next day for lysis and plaque formation. The titers of the phages were determined by mixing 100 μL of phage dilution with 200 μL of ON bacterial culture in 4 mL soft agar and overlaid onto BHI agar plates. The plates were incubated ON at 37°C, and plaques were counted the next day. Double-layer titration was used to determine the EOP, using the titer of propagation strain 8325-4 as a reference and where EOP was calculated by dividing the titer on each strain by the titer on the propagation strain. Turbidity reduction assays were performed in 96-well plates (CytoOne, multiwell plate with lid) or in glass tubes for viable phage and bacterial counts. ON cultures were diluted 20×, and phages were added to an MOI of 1.0. The plates were incubated at 37°C with shaking, and the OD_600_ was measured every 5 min for 24 h in a TECAN infinite 200Pro, Tecan i-control 1.10.4.0. For viable counts, bacteria were serially diluted in NaCl 0.9% and enumerated. Phage concentration was determined with dilution spot assays. All experiments here and for other *in vitro* experiments were performed in triplicate.

### Adsorption assays.

Phage adsorption was measured similarly to previous studies (30, 59). Briefly, ON cultures were diluted to a concentration of approximately 1 × 10^8^ CFU/mL in fresh media, to which phage was added at an MOI of 0.001 and incubated at 37°C with shaking. Aliquots of 1 mL were sampled at 5 and 20 min and centrifuged for 5 min at room temperature (RT), and the supernatant was used for phage titration. Free phage concentrations were also determined at later time points to follow the infection process. Two-tailed *t* tests were used to determine significance.

### Lysostaphin assays.

Bacteria were grown to an OD_600_ of ~0.45, at which point they were pelleted and their OD_600_ values were adjusted to 1.0 in NaCl 0.9%. Two-fold serial dilutions of lysostaphin in phosphate-buffered saline (PBS) were prepared in a 96-well plate, to which 150 μL of bacterial suspension was added. The OD_600_ was measured over time (EL808 OD microplate reader, Gen5 software, BioTek).

### qPCR.

A protocol to detect intracellular phage DNA was adapted from the work of Fernandes et al. (33). Bacteria were grown to an OD_600_ of 0.3 to 0.4 and infected with phage K at an MOI of 0.1. Aliquots of 10 mL were removed from the continuous culture at indicated time points and centrifuged for 5 min at 8,000 rpm at RT. The pellets were resuspended in 20 mL of buffer (0.1 M Tris-HCl, 0.1 M NaCl, 50 mM EDTA), incubated at 80°C for 15 min in a water bath, and then centrifuged. Pellets were suspended in 100 μL of TBT buffer (100 mM Tris-HCl, 100 mM NaCl, 100 mM MgCl_2_) with 250 μg/mL proteinase K. This solution was left for 1 h at 55°C with shaking, and the proteinase K deactivated for 15 min at 70°C. After cooling, 0.5 μL of benzonase nuclease (250 units/μL; Sigma-Aldrich) was added and incubated for 1 h at 37°C before washing twice in Tris-EDTA (TE; 10 mM Tris-HCl, 1 mM EDTA, pH 8) buffer and centrifuging for 10 min at top speed. The ability of this protocol to remove unadsorbed phage and degrade free phage DNA was verified by PCR on similarly treated phage suspensions.

All bacterial DNA extractions were performed following a protocol adapted from Bae et al. (60) using Promega solutions as follows: 3 mL ON cultures were centrifuged for 1 min at 1,000 rpm, and the pellet was resuspended in 50 μL TE supplemented with 2 μL lysostaphin (20 mg/mL). Suspensions were then incubated ON at 37°C, and the following day 300 μL of nuclei lysis solution was added (Promega, USA). Extractions were vortexed and incubated for at least 10 min at 80°C and then cooled to RT before 2 μL RNase (10 mg/mL) were added. Extractions were vortexed again and incubated for at least 30 min at 37°C before the addition of 100 μL protein precipitation solution followed by subsequent incubation on ice for 5 min. The samples were then centrifuged at top speed for 10 min at 4°C. This step was repeated until the samples were clear. Supernatants were transferred to a new Eppendorf tube containing 300 μL of RT isopropanol, inverted several times to mix, and centrifuged for an additional 10 min at top speed at 4°C. Supernatants were then gently discarded, and 750 μL of 70% ethanol was added to the tubes. After another round of centrifugation, the supernatant was gently discarded, and the pellet was dried at 37°C before it was resuspended in 20 μL TE and incubated ON at 4°C. The following day, the samples were incubated 1 h at 65°C before measuring the DNA concentration using a nanodrop, and the concentrations were then verified with gel electrophoresis.

A 100-bp region on the genome of phage K between positions 9388 and 9487 (accession number KF766114; k1_fw, 5′-CACGACGTTGTAAAACGA; k1_rv, 5′-CCTGTGTGAAATTGTTATCC) was amplified from phage K. A 139-bp region of the 16SrRNA from PC3* was amplified using the primers PP060 (5′-TTAGATACCCTGGTAGTCCAC) and PP61 (5′-CCCGTCAATTCCTTTGAGTTT) and used as a control for cell number (kindly provided by Philippe Piccardi, UNIL). Amplicons were ligated into the pGEM-T vector (Promega) and transformed into Escherichia coli DH5α (Amp-resistant, XGAL), and inserts were verified by sequencing, before using amplicons to create a standard curve. qPCRs were prepared using 1 μL template DNA, previously adjusted to 1 ng/μL, with 0.2 μL of forward and reverse primers (10 μM), 3 μL PURE water, and 4 μL KAPA SYBR FAST (Sigma-Aldrich,) qPCR Master mix. The reactions were amplified in a Rotor-Gene Q machine (Qiagen) with an imported standard curve and analyzed with corresponding software from supplier to calculate the average copy number. An initial cycle of 3 min at 95°C preceded 40 cycles of 95°C for 3 s and 60°C for 20 s.

### Transmission Electron Microscopy (TEM).

Bacteria and phage cultures were concentrated and fixed in 2.5% glutaraldehyde (EMS) in phosphate buffer (PB; 0.1 M, pH 7.4; Sigma) for 1 h at RT prior to high pressure freezing using an HPF Compact 02 (Wohlwend GmbH). The samples were then cryosubstituted in an AFS2 (Leica Mikrosysteme GmbH) in 2% osmium tetroxide (EMS) in pure acetone (Sigma), from −90 to −30°C over 90 h and then washed twice in 100% acetone at −30°C for 1 h. This was followed by infiltration in Epon (Sigma) at graded concentrations of Epon (Epon 1/3 acetone for 2.5 h from −30 to 0°C; Epon 3/1 acetone for 2.5 h from 0 to 20°C; Epon 1/1 for 2.5 h at 20°C). The samples were then put into molds and polymerized for 48 h at 60°C.

Ultrathin sections of 50 nm were cut on a Leica Ultracut (Leica Mikrosysteme GmbH) and retrieved on a copper-slot grid (2 × 1 mm; EMS) coated with a polystyrene film (Sigma). Sections were poststained with 4% uranyl acetate (Sigma) in H_2_O for 10 min; rinsed several times with H_2_O, which was followed by Reynolds lead citrate in H_2_O (Sigma) for 10 min; and rinsed several times with H_2_O.

Micrographs were taken with a transmission electron microscope Philips CM100 (Thermo Fisher Scientific) at an acceleration voltage of 80 kV with a TVIPS TemCam-F416 digital camera (TVIPS GmbH). Large montage alignments were performed using Blendmont command-line program from the IMOD software package (61). Computer visualization of three-dimensional image data and image analysis was done using IMOD. Cell wall thickness was measured with Fiji for ~10 cells/condition, as described previously (62, 63).
