# Supplementary material for: Antibiotic Exposure Leads to Reduced Phage Susceptibility in Vancomycin Intermediate Staphylococcus aureus (VISA)
Source: Antimicrob Agents Chemother. 2022 Jun 16;66(7):e02247-21. doi: 10.1128/aac.02247-21 (PMC9295574; doi:10.1128/aac.02247-21)

Supplementary material

Supplementary Table 1: VRSA strains and phage sensitivity. All strains were obtained from BEI resources and had vancomycin MIC values >16 ug/mL. Similar to the method for inducing the VISA phenotype, VRSA strains were passed with or without vancomycin (8 ug/mL) in BHI for 10 days, at which time they were tested for phage susceptibility. Vancomycin passaging did not result in a change in phage sensitivity for any strain and therefore, phage sensitivity shown in the table is representative for that strain with or without vancomycin exposure.

	**Strain**	**Phage**	**Location, Year**	**Isolation site**
1	HIP11714	S	MC 2002	Infected catheter exit
2	HIP11983	R	PA 2002	Infected plantar ulcer
3	HIP13419	R	NY 2004	Nephrostomy tube exit
4	HIP14300	S	MC 2005	Gangrenous toe wound
5	HIP15178	S	MC 2005	Surgical site infection
6	AIS2006032	S	MC 2005	Infected plantar ulcer
7	AIS2006045	S	MC 2006	Necrotizing fasciitis upper limb
8	71080	R	MC 2007	Infected toe wound
9	AIS080003	R	MC 2007	Infected plantar foot wound
10	AIS100505	S	MC 2009	Infected plantar foot wound
11	AID1001123	S	DE 2010	Wound drainage from PJI

S Sensitive; R Resistant; All strains were isolated from states in the US: MC Michigan; PA Pennsylvania; NY New York; DE Delaware; PJI prosthetic joint infection

Supplementary Figure 1: Plaque size difference for phage K between strains (VSSA 8325-4 and hVISA Mu3) with different vancomycin profiles.

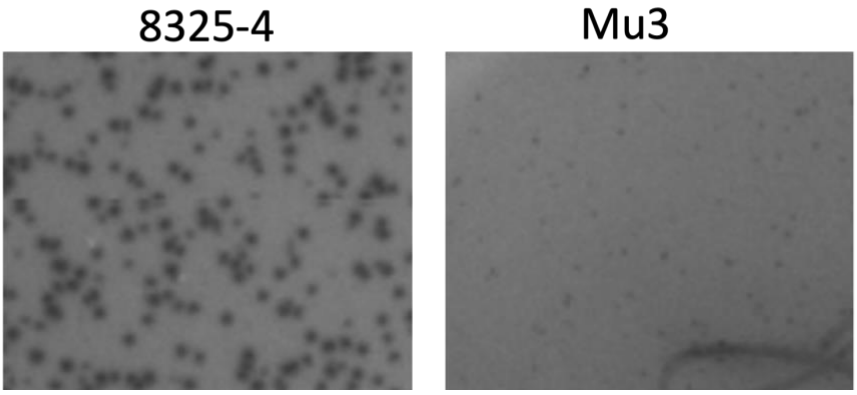

Supplementary Figure 2: Spot assays on parental strains of PC3*van-/+ and EOP values.

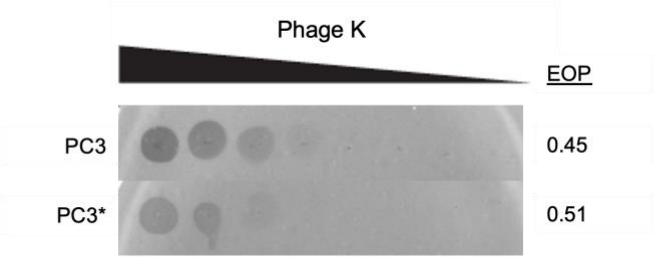

Supplementary Figure 3: Infection time course of viable phage counts on PC3*van-/+ over time with an initial MOI of 0.01. Aliquots of 1 ml were sampled at specified times, centrifuged at room temperature (RT) for 10 min and filtered (0.44 µm) for phage titration.

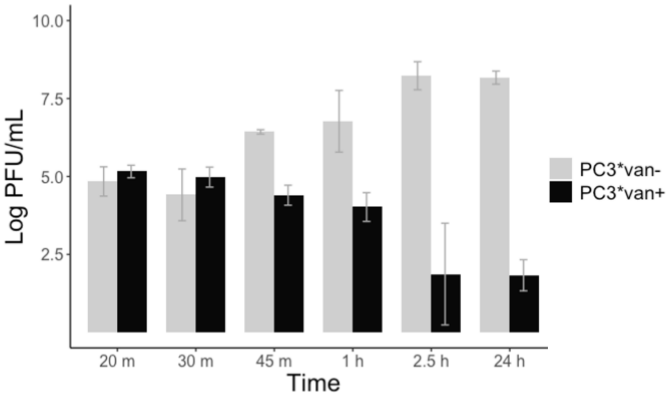

Supplementary Figure 4: EM montage of phage-infected cultures 20 min post addition of phage analysed with IMOD using the Stereology plugin. Gridlines measured 500x500 nm and cells were marked as “Bacteria with adsorbed phage, EMPTY CAPSID” (green), “Bacteria with adsorbed phage, FULL CAPSID” (yellow), “Bacteria no adsorbed phage/broken tail visible” (shades of blue). Both the cell counts and volume density did not show a significant difference for empty/full capsids between PC3*van+ and PC3*van- phage-infected cultures (chi-square test).

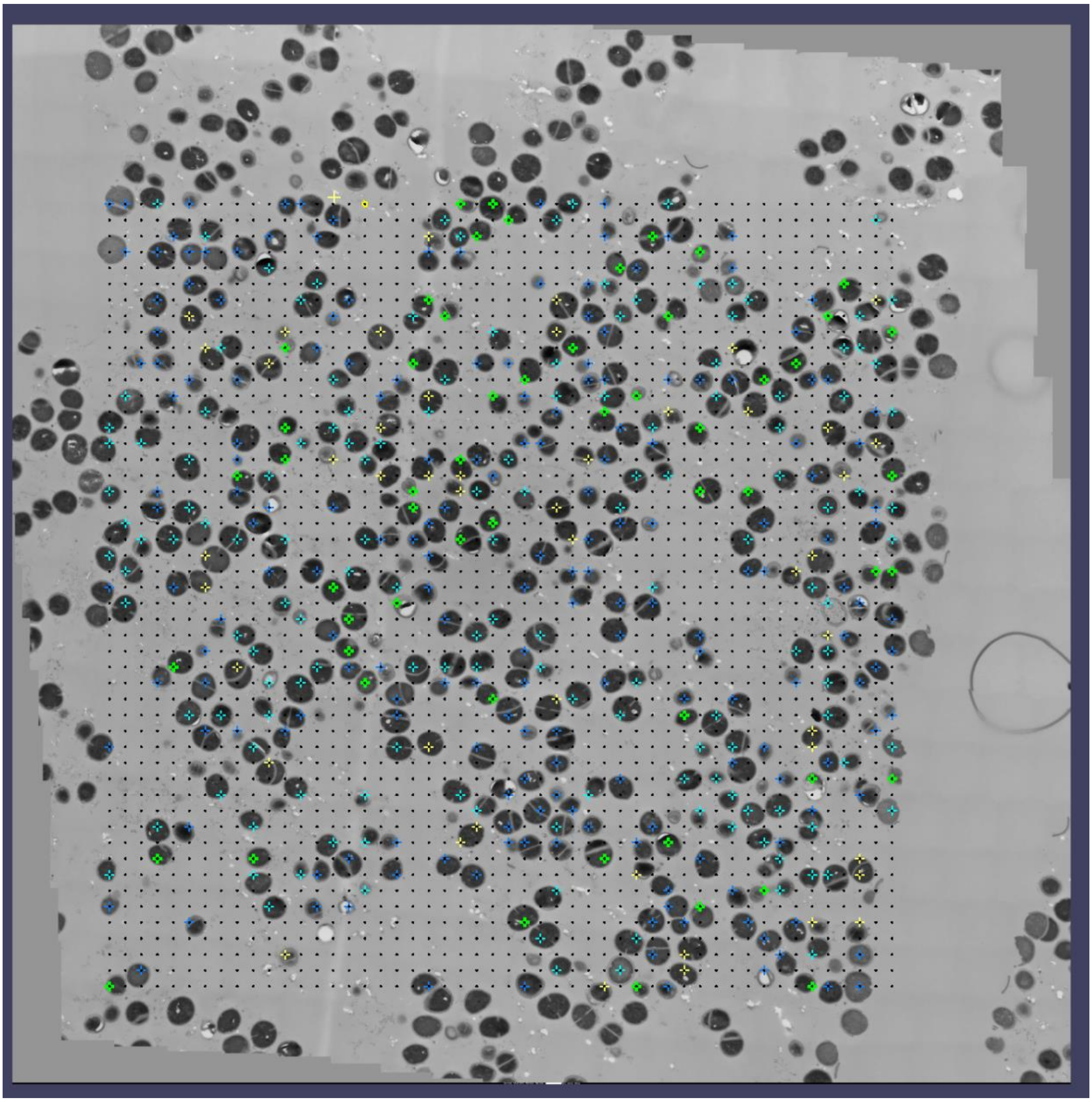

Supplement: Supplemental file 1 — Table S1 and Fig. S1 to S4. Download aac.02247-21-s0001.pdf, PDF file, 0.4 MB [file aac.02247-21-s0001.pdf]
